# Supplementary material for: Heat adaptation of phage T7 under an extended genetic code
Source: Virus Evol. 2021 Dec 1;7(2):veab100. doi: 10.1093/ve/veab100 (PMC8923235; doi:10.1093/ve/veab100)
Supplement: veab100_Supp [file veab100_supp.zip › Sequencing_Analysis.zip!/Sequencing_Analysis/S2/evidence/JC_SIDE_1_65.html]

BRESEQ :: Evidence


| New junction evidence | | | | | | | | | | | |
| --- | --- | --- | --- | --- | --- | --- | --- | --- | --- | --- | --- |
|  | | seq id | position | reads (cov) | reads (cov) | score | skew | freq | annotation | gene | product |
| \* | ? | NC\_001604.1 | = 13586 | 0 (0.000) | 46 (0.010) | 20/592 | 45.7 | 50.3% | intergenic (–/–) | *–/–* | –/– |
|  | ? | NC\_001604.1 | 14356 = | 91 (0.020) |  |  |  |  | intergenic (–/–) | *–/–* | –/– |
| Rejected: Coverage evenness skew score above cutoff. | | | | | | | | | | | |

`CCAGACGACTACGAGAAGTTTCGCTGGAGAGTCCCATTCTAATACGACTCACTAAAGGAGACACACCATGTTCAAACTGATTAAGAAGTTAGGCCAACTGCTGGTTCGTATGTACAACGTGGAAGCCAAGCGACTGAACGATGAGGCTCGTAAAGAGGCCACACAGTCACGCGCTCTGGCGATTCGCTCCAACGAACTGGCTGACAGTGCATCCACTAAAGTTACCGAGGCTGCCCGTGTGGCAAACCAAGCTCAACAGCTTTCCAAATTCTTTGAGTAATCAAACAGGAGAAACCATTATGTCTAACGTAGCTGAAACTATCCGTCTATC  >  NC_001604.1/13285‑13615`  

                                                                                                                                                                                                                                                                                                             `|`                               
`ccAGACGACTACGAGAAGTTTCGCTGGAGAGTCCCATTCTAATACGACTCACTAAAGGAGACACA‑CATGTTCAAACTGATTAAGAAGTTAGGCCAACTGCTGGTTCGTATGTACAACGTGGAAGCCAAGCGACTGAACGATGAGGCTCGTAAAGAGGCCACACAGTCACGCGCTCTGGCGATTCGCTCCAACGAACTGGCTGACAGTGCATCCACTAAAGTTACCGAGGCTGCCCGTGTGGCAAACCAAGCTCAACAGCTTTCCAAATTCTTTGAGTAATCAAACAGGAGAAACCATTATg                               <  1:684772/301‑1 (MQ=255)`  
 `cAGACGACTACGAGAAGTTTCGCTGGAGAGTCCCATTCTAATACGACTCACTAAAGGAGACACACCATGTTCAAACTGATTAAGAAGTTAGGCCAACTGCTGGTTCGTATGTACAACGTGGAAGCCAAGCGACTGAACGATGAGGCTCGTAAAGAGGCCACACAGTCACGCGCTCTGGCGATTCGCTCCAACGAACTGGCTGACAGTGCATCCACTAAAGTTACCGAGGCTGCCCGTGTGGCAAACCAAGCTCAACAGCTTTCCAAATTCTTTGAGTAATCAAACAGGAGAAACCATTATg                               <  1:7049/301‑1 (MQ=255)`  
  `aGACGACTACGAGAAGTTTCGCTGGAGAGTCCCATTCTAATACGACTCACTAAAGGAGACACACCATGTTCAAACTGATTAAGAAGTTAGGCCAACTGCTGGTTCGTATGTACAACGTGGAAGCCAAGCGACTGAACGATGAGGCTCGTAAAGAGGCCACACAGTCACGCGCTCTGGCGATTCGCTCCAACGAACTGGCTGACAGTGCATCCACTAAAGTTACCGAGGCTGCCCGTGTGGCAAACCAAGCTCAACAGCTTTCCAAATTCTTTGAGTAATCAAACAGGAGAAACCATTATga                              <  1:504778/301‑2 (MQ=255)`  
  `aGACGACTACGAGAAGTTTCGCTGGAGAGTCCCATTCTAATACGACTCACTAAAGGAGACACACCATGTTCAAACTGATTAAGAAGTTAGGCCAACTGCTGGTTCGTATGTACAACGTGGAAGCCAAGCGACTGAACGATGAGGCTCGTAAAGAGGCCACACAGTCACGCGCTCTGGCGATTCGCTCCAACGAACTGGCTGACAGTGCATCCACTAAAGTTACCGAGGCTGCCCGTGTGGCAAACCAAGCTCAACAGCTTTCCAAATTCTTTGAGTAATCAAACAGGAGAAACCATTATga                              <  1:559612/301‑2 (MQ=255)`  
   `gggcgcggatatgtgtataagcgtcaggTCCCATTCTAATACGACTCACTAAACGAGACACACCGTGTTCAAACGTATTAAGAAGTTAGGCCAACTGCTTGTTCGTATGTACAACGTGGAAGCCAAGCGACTGAACGATGAGGCTCGTAAAGAGGCCACACAGCCACGCGCTCTGGCGATTCGCTCCAACGAACTGGCTGACAGTGCATCCACTAAAGTTACCGAGGCTGCCCGTGTGGCAAACCAAGCTCAACAGCTTTCCAAATTCTTTGAGTAATCAAACAGGAGAAACCATTATgat                             <  1:347067/274‑3 (MQ=255)`  
   `gacgacTACGAGAAGTTTCGCTGGAGAGTCCCATTCTAATACGACTCACTAAAGGAGACACACCATGTTCAAACTGATTAAGAAGTTAGGCCAACTGCTGGTTCGTATGTACAACGTGGAAGCCAAGCGACTGAACGATGAGGCTCGTAAAGAGGCCACACAGTCACGCGCTCTGGCGATTCGCTCCAACGAACTGGCTGACAGTGCATCCACTAAAGTTACCGAGGCTGCCCGTGTGGCAAACCAAGCTCAACAGCTTTCCAAATTCTTTGAGTAATCAAACAGGAGAAACCATTATgat                             <  1:101506/301‑3 (MQ=255)`  
   `gacgacTACGAGAAGTTTCGCTGGAGAGTCCCATTCTAATACGACTCACTAAAGGAGACACACCATGTTCAAACTGATTAAGAAGTTAGGCCAACTGCTGGTTCGTATGTACAACGTGGAAGCCAAGCGACTGAACGATGAGGCTCGTAAAGAGGCCACACAGTCACGCGCTCTGGCGATTCGCTCCAACGAACTGGCTGACAGTGCATCCACTAAAGTTACCGAGGCTGCCCGTGTGGCAAACCAAGCTCAACAGCTTTCCAAATTCTTTGAGTAATCAAACAGGAGAAACCATTATgat                             <  1:292489/301‑3 (MQ=255)`  
   `gacgacTACGAGAAGTTTCGCTGGAGAGTCCCATTCTAATACGACTCACTAAAGGAGACACACCATGTTCAAACTGATTAAGAAGTTAGGCCAACTGCTGGTTCGTATGTACAACGTGGAAGCCAAGCGACTGAACGATGAGGCTCGTAAAGAGGCCACACAGTCACGCGCTCTGGCGATTCGCTCCAACGAACTGGCTGACAGTGCATCCACTAAAGTTACCGAGGCTGCCCGTGTGGAAAACCAAGCTCAACAGCTTTCCAAATTCTTTGAGTAATCAAACAGGAGAAACCATTATgat                             >  1:154483/1‑299 (MQ=255)`  
   `acagacTACGAGAAGTTTCGCTGGAGAGTCCCATTCTAATATGACTCACTAAAGGAGACACACCATGTTCAAACTGATTAAGAAGTTAGGCCAACTGCTGGTTCGTATGTACAACGTGGAAGCCAAGCGACTGAACGATGAGGCTCGTAAAGAGGCCACACAGTCACGCGCTCTGGCGATTCGCTCCAACGAACTGGCTGACAGTGCATCCACTAAAGTTACCGAGGCTGCCCGTGTGGCAAACCAAGCTCAACAGCTTTCCAAATTCTTTGAGTAATCAAACAGGAGAAACCATTATgat                             <  1:192070/298‑3 (MQ=255)`  
       `acTACGAGAAGTTTCGCTTGAGAGTCCCATTCTAATACGACTCACTAAAGGAGACACACCATGTTCAAACTGATTAAGAAGTTAGGCCAACTGCTGGTTCGTATGTACAACGTGGAAGCCAAGCGACTGAACGATGAGGCTCGTAAAGAGGCCACACAGTCACGCGCTCTGGCGATTCGCTCCAACGAACTGGCTGACAGTGCATCCACTAAAGTTACCGAGGCTGCCCGTGTGGCAAACCAAGCTCAACAGCTTTCCAAATTCTTTGAGTAATCAAACAGGAGAAACCATTATgctctgt                         >  1:493058/1‑295 (MQ=255)`  
       `acTACGAGAAGTTTCGCTGGAGAGTCCCATTCTAATACGACTCACTAAAGGAGACACACCATGTTCAAACTGATTAAGAAGTTAGGCCAACTGCTGGTTCGTATGTACAACGTGGAAGCCAAGCGACTGAACGATGAGGCTCGTAAAGAGGCCACACAGTCACGCGCTCTGGCGATTCGCTCCAACGAACTGGCTGACAGTGCATCCACTAAAGTTACCGAGGCTGCCCGTGTGGCAAACCAAGCTCAACAGCTTTCCAAATTCTTTGAGTAATCAAACAGGAGAAACCATTATgatcgtt                         >  1:231170/1‑295 (MQ=255)`  
       `acTACGAGAAGTTTCGCTGGAGAGTCCCATTCTAATACGACTCACTAAAGGAGACACACCATGTTCAAACTGATTAAGAAGTTAGGCCAACTGCTGGTTCGTATGTACAACGTGGAAGCCAAGCGACTGAACGATGAGGATCGTAAAGAGGCCACACAGTCACGCGATCTGGCGATTCGCTCCAACGAACTGGCTGACAGTGCATCCACTAAAGTTACCGAGGCTGCCCGTGTGGCAAACCAAGCTCAACAGCTTTCCAAATTCTTTGAGTAATCAAACAGGAGAAACCATTATgatcgtt                         >  1:825831/1‑295 (MQ=255)`  
         `tACGAGAAGCTTCGCTGGAGAGTCCCATTCTAATACGACTCACTAAAGGAGACACACCATGTTCAAACTGATTAAGAAGTTAGGCCAACTGCTGGTTCGTATGTACAACGTGGAAGCCAAGCGACTGAACGATGAGGCTCATAAAGAGGCCACACAGTCACGCGCTCTGGCGATTCGCTCCAACGAACTGGCTGACAGTGCATCCACTAAAGTTACCGAGGCTGCCCGTGTGGCAAACCAAGCTCAACAGCTTTCCAAATTCTTTGAGTAATCAAACAGGAGAAACCATTATgatcgtttt                       >  1:708280/1‑293 (MQ=255)`  
          `aCGAGAAGTTTCGCTGGAGAGTCCCATTCTAATACGACTCACTAAAGGAGACACACCATGTTCAAACTGATTAAGAAGTTAGGCCAACTGCTGGTTCGTATGTACAACGTGGAAGCCAAGCGACTGAACGATGAGGCTCGTAAAGAGGCCACACAGTCACGCGCTATGTCGATTCGCTCCAACGAACTGGCTGACAGTGCATCCACTAAAGTTACCGAGGCTGCCCGTGTGGCAAACCAAGCTCAACAGCTTTCCAAATTCTTTGAGTAATCAAACAGGAGAAACAATTATgaccgtttct                      >  1:496043/1‑292 (MQ=255)`  
                    `tCGCTGGAGAGTCCCATTCTAATACGACTCACTAAAGGAGACACACCATGTTCAAACTGATTAAGAAGTTAGGCCAACTGCTGGTTCGTATGTACAACGTGGAAGCCAAGCGACTGAACGATGAGGCTCGTAAAGAGGCCACACAGTCACGCGCTCTGGCGATTCGCTCCAACGAACTGGCTGACAGTGCATCCACTAAAGTTACCGAGGCTGCCCGTGTGGCAAACCAAGCTCAACAGCTTTCCAAATTCTTTGAGTAATCAAACAGGAGAAACCATTATgatcctgtctcttatacaca            >  1:163943/1‑282 (MQ=255)`  
                              `gTCCCATTCTAATACGACTCACTAAAGGAGACACACCATGTTCAAACTGATTAAGAAGTTAGGCCAACTGCTGGTTCGTATGTACAACGTGGAAGCCAAGCGACTGAACGATGAGGCTCGTAAAGAGGCCACACAGTCACGCGCTCTGGCGATTCGCTCCAACGAACTGGCTGACAGTGCATCCACTAAAGTTACCTAGGCTGCCCGTGTGGCAAACCAAGCTCAACAGCTTTCCAAATTCTTTGAGTAATCAAACAGGAGAAACCATTATgatcgctgtctcttatacacatctccgagc  >  1:160231/1‑272 (MQ=255)`  

                                                                                                                                                                                                                                                                                                             `|`                               
`CCAGACGACTACGAGAAGTTTCGCTGGAGAGTCCCATTCTAATACGACTCACTAAAGGAGACACACCATGTTCAAACTGATTAAGAAGTTAGGCCAACTGCTGGTTCGTATGTACAACGTGGAAGCCAAGCGACTGAACGATGAGGCTCGTAAAGAGGCCACACAGTCACGCGCTCTGGCGATTCGCTCCAACGAACTGGCTGACAGTGCATCCACTAAAGTTACCGAGGCTGCCCGTGTGGCAAACCAAGCTCAACAGCTTTCCAAATTCTTTGAGTAATCAAACAGGAGAAACCATTATGTCTAACGTAGCTGAAACTATCCGTCTATC  >  NC_001604.1/13285‑13615`

  

Alignment Legend

Aligned base mismatch/match (shaded by quality score): `ATCG/ATCG` `< 3 ≤` `ATCG/ATCG` `< 18 ≤` `ATCG/ATCG` `< 30 ≤` `ATCG/ATCG` `< 37 ≤` `ATCG/ATCG` `< 38 ≤` `ATCG/ATCG`

Unaligned base: `atcg`    Masked matching base: `atcg`    Alignment gap: `‑`    Deleted base: `‑`

Reads not counted as support for junction

read\_name Not counted due to insufficient overlap past the breakpoint.

read\_name Not counted due to not crossing MOB target site duplication.
